# Two Novel Lytic Bacteriophages Infecting *Enterococcus* spp. Are Promising Candidates for Targeted Antibacterial Therapy

**DOI:** 10.3390/v14040831

**Published:** 2022-04-16

**Authors:** Pavel V. Tkachev, Ivan M. Pchelin, Daniil V. Azarov, Andrey N. Gorshkov, Olga V. Shamova, Alexander V. Dmitriev, Artemiy E. Goncharov

**Affiliations:** 1 Scientific and Educational Center “Molecular Bases of Interaction of Microorganisms and Human” of the WCRC “Center for Personalized Medicine”, Institute of Experimental Medicine, 197022 Saint Petersburg, Russia; arcella.oraia@gmail.com (I.M.P.); denazarov.da@gmail.com (D.V.A.); oshamova@yandex.ru (O.V.S.); admitriev10@yandex.ru (A.V.D.); 2 Smorodintsev Research Institute of Influenza, Ministry of Health of the Russian Federation, 197376 Saint Petersburg, Russia; angorsh@yahoo.com; 3 Laboratory of Pathomorphology, Almazov National Research Centre, 197341 Saint Petersburg, Russia

**Keywords:** bacteriophage, *Enterococcus* virus, phage therapy

## Abstract

The rapid emergence of antibiotic resistance is of major concern globally. Among the most worrying pathogenic bacteria are vancomycin-resistant enterococci. Phage therapy is a highly promising method for controlling enterococcal infections. In this study, we described two virulent tailed bacteriophages possessing lytic activity against *Enterococcus faecalis* and *E. faecium* isolates. The SSsP-1 bacteriophage belonged to the *Saphexavirus* genus of the *Siphoviridae* family, and the GVEsP-1 bacteriophage belonged to the *Schiekvirus* genus of *Herelleviridae*. The genomes of both viruses carried putative components of anti-CRISPR systems and did not contain known genes coding for antibiotic-resistance determinants and virulence factors. The conservative arrangement of protein-coding sequences in *Saphexavirus* and *Schiekvirus* genomes taken together with positive results of treating enterococcal peritonitis in an animal infection model imply the potential suitability of GVEsP-1 and SSsP-1 bacteriophages for clinical applications.

## 1. Introduction

The bacteria of the genus *Enterococcus* form a part of the human microbiome. In healthy individuals, they reside mainly in the gastrointestinal tract and participate in food digestion [1]. However, adapted to the hospital environment, vancomycin-resistant and multidrug-resistant *Enterococcus* strains are frequent cause of lethal system blood infections in comparison with antibiotic-sensitive strains [2,3,4]. In leukemia patients, vancomycin-resistant enterococcal infections have associated mortality rates estimated at 57% [5]. Infections caused by antibiotic-resistant strains of enterococci harm individuals and cause damage to society as a whole [6]. The two *Enterococcus* species encountered most often in multidrug-resistant nosocomial infections are *E.* *faecium* and E. faecalis [7,8]. Of these two species, drug-resistant *E.* *faecium* is on the ESKAPE list compiled by the Infectious Diseases Society of America, highlighting the utmost importance of developing alternative therapies against this pathogen [9]. Moreover, taking into account a number of factors, including mortality, health-care burden and others, the World Health Organization considered the development of effective drugs against vancomycin-resistant strains of *E. faecium* to be a high priority [10].

In recent decades, the decreasing rates of progress in the discovery of antibiotics have promoted research interest in bacteriophage therapy [11,12]. Though phage therapy has not become mainstream yet, there are a number of well-described successfully treated cases of surgical infections at present [13,14,15]. Enterococcal viruses are also promising agents for treating oral infections [16] and intestinal dysbiosis [17]. Among their potential uses is development for bio-sanitizing formulations [18]. Since the application of therapeutic bacteriophages is naturally limited by their narrow host ranges and host resistance [19], there is a continuous need for improvements in the spectrum of available bacteriophages.

Both the advantage and disadvantage of phage therapy as compared with antibiotics is its selectivity [20]. Bacteriophages usually target a limited number of strains within bacterial species. Cross-genus tropism of some phages is known, but the examples are quite rare [21]. There are several requirements for therapeutic bacteriophages. The list includes natural origin of a bacterial virus, known taxonomic identity, absence of harmful genetic determinants, absence of transducing activity and high in vitro efficacy [22]. Here we describe two novel virulent *Enterococcus* bacteriophages. We analyze their host range, morphology, whole-genome sequences and potential to eradicate a model infection in mice.

## 2. Materials and Methods

### 2.1. Bacteriophage Isolation and Preparation

Four *E. faecalis* strains were used for viral propagation in vitro and in vivo experiments (Table 1). Our bacteriophages originated from fecal samples and river water. The isolation of bacterial viruses was performed according to slightly modified classical protocols [23,24]. The protocol for phage isolation from feces included the initial step of resuspension; all other steps were similar. The fecal sample was resuspended by vortexing in sterile SM buffer. The SM buffer consisted of 10 mM NaCl, 10 mM MgSO_4_, 50 mM Tris-HCl pH 7.5 and 0.05% gelatin. In the next step, 15 mL of the diluted fecal sample or river water were centrifuged at 4500× *g* for 5 min and filtered through 0.45 µm pore syringe filters with PES membranes (Jet Biofil, YongHe Development Zone, Guangzhou, China). One milliliter of filtrate was supplied with 200 µL 5× Todd-Hewitt broth (HiMedia Laboratories, Mumbai, India). The sample of river water was also supplemented with CaCl_2_ and MgSO_4_ to obtain equal final concentrations at 10 mM. Then, the samples were inoculated with 20 µL of 18–24 h bacterial cultures and incubated at 30 °C overnight. The resulting lysate was centrifuged at 4500× *g* and filtered through 0.45 µm pore syringe filters. The presence of bacteriophages was revealed by spot test with a series of 10-fold dilutions. The pure viral cultures were obtained by triple propagation in sensitive bacterial strains.

Phage stocks used in all described here in vitro and in vivo experiments and DNA sequencing were prepared in the following way. The precipitation of viral particles was performed by supplementing 50 mL of viral cultures with PEG6000 up to 10% *w*/*v* and NaCl to a final concentration of 1 M and mixed, then kept at 4 °C overnight. This was followed by centrifugation at 5000× *g* for 60 min. The resulting pellet was resuspended in SM buffer. The solution was filtered with the use of 0.45 µm pore syringe filters.

### 2.2. Transmission Electron Microscopy

For transmission electron microscopy, the studied viral stocks were applied to copper grids (300 mesh, Sigma-Aldrich, St. Louis, MO, USA), coated with a collodion film substrate. After adsorption of particles from suspension to the supporting film for 1–2 min, the meshes were washed twice with distilled water. Further, negative contrasting of the samples was carried out for 1–2 min in sodium salt of phosphoric–tungstic acid, 2% aqueous solution (Sigma-Aldrich), pH 7.2. Afterwards, the meshes were dried and examined on a transmission electron microscope JEM 1011 (JEOL, Tokyo, Japan). Instrumental magnification ranged from 50,000× to 200,000×. Electron micrographs were obtained using a Morada high-resolution digital camera (Olympus, Tokyo, Japan). In each case, 20–30 fields of view were examined. Phage size measurements were calculated from TEM images (GVEsP-1, *n* = 10; SSsP-1, *n* = 4). The variability of phage dimensions was assessed by calculating the standard deviations of the mean values.

### 2.3. In Vitro Efficacy

The length of the latent period was assessed by one-step growth experiments, performed as described by Adams [23], with few modifications. To determine the optimal multiplicity of infection (MOI) of phages, *E. faecalis* strains Serg and CCUG 52538 were infected with SSsP-1 and GVEsP-1 phages, respectively, with different MOIs (0.001, 0.01, 0.1, 1, 10, 100). After six hours of incubation, MOIs of propagated phages were counted in supernatant using the double-layer agar method. For one step growth experiment, 0.1 mL of phage stock (3 × 10^8^ PFU) was mixed with 0.9 mL of bacterial test culture (3 × 10^9^ CFU) (MOI = 0.01). After 10 min of incubation at 37 °C, the mixture was centrifuged at 10,000× *g* for 5 min, the supernatant was discarded and the pellet was resuspended in a fresh 10 mL of Todd-Hewitt broth. Then, portions of 0.1 mL of broth were taken every 5 min and titrated against a host culture. The experiment was performed in triplicate. To calculate the burst size of each bacteriophage, we used the method described in reference [25].

### 2.4. Determining the Host Range of Viruses

Host range was determined with the use of an in-house collection of 82 bacterial strains (Table S1). The list of *Enterococcus* spp. strains included *E. faecalis* (*n* = 39), *E. faecium* (*n* = 23), *E. casseliflavus* (*n* = 1), *E. durans* (*n* = 1), *E. hirae* (*n* = 1) and *E. gallinarum* (*n* = 2). We also tested the phages with *Staphylococcus aureus* (*n* = 5), *S. epidermidis* (*n* = 1), *Streptococcus agalactiae* (*n* = 3), *S. pyogenes* (*n* = 4), *Bifidobacterium longum* (*n* = 1) and *Escherichia coli* (*n* = 1) isolates. Bacterial species identification was carried out using a BactoSCREEN MALDI-TOF MS system (Lytech, Moscow, Russia). All *Enterococcus* and *Streptococcus* strains were grown on Todd-Hewitt broth and Todd-Hewitt agar at 37 °C (HiMedia Laboratories, Mumbai, India). *Staphylococcus* spp. strains were grown on Mannitol salt broth and Mannitol salt agar (SRCAMB, Obolensk, Russia). The *Escherichia coli* strain was grown on LB agar and LB broth (BioFroxx, Einhausen, Germany). The *Bifidobacterium longum* strain was tested on Bifidum agar and Bifidum broth (SRCAMB) under anaerobic conditions. Host range was determined by spot test with eight serial 10-fold dilutions of phage stock on a bacterial lawn.

### 2.5. DNA Isolation and Whole-Genome Sequencing

To isolate viral DNA, the standard phenol/chloroform DNA extraction protocol was used [26]. The phage genome sequences were obtained with the use of the Illumina MiSeq platform (Illumina, San Diego, CA, USA). For GVEsP-1, library preparation was carried out with a Nextera XT DNA Library Preparation Kit (Illumina), resulting in paired-end 300 bp reads. The library for SSsP-1 genome was obtained with the use of a NEBNext Ultra II DNA Library Prep Kit (New England Biolabs, Ipswich, MA, USA) with an average read size of 350 bp. The raw reads were quality controlled using FastQC v0.11 (https://www.bioinformatics.babraham.ac.uk/projects/fastqc/, accessed on 1 May 2020). The viral genomes were assembled de novo by SPAdes 3.13.0 [27]. The nucleotide sequences of the genomes were deposited in the NCBI Nucleotide database. The GenBank/ENA/DDBJ accession number for GVEsP-1 is MZ333462. The accession number for the SSsP-1 genome is MZ333457.

### 2.6. Bioinformatic Analysis

We visualized the whole-genome structure of the two studied viruses using CGView 1.7 Server [28]. The genetic structures of the genera *Schiekvirus* and *Saphexavirus* were visualized by phylogenetic network analysis. For each of the two studied viruses, the sequences of five protein-coding genes were used for BLASTN searches against the NCBI Nucleotide database. The marker genes were supplemented with genes having the widest possible taxonomic coverage. Selected GVEsP-1 sequences coded for the baseplate assembly protein, capsid and scaffold protein, DNA helicase, DNA polymerase and major capsid protein. The studied SSsP-1 sequences included the genes coding for the minor capsid protein, minor structural protein, portal protein, replicative DNA helicase and terminase large subunit. The concatenated alignments of these genes were used to calculate NeighborNet phylogenetic networks in SplitsTree 4.14.2 software [29].

To analyze protein-coding gene synteny in *Schiekvirus* spp. and *Saphexavirus* spp., we downloaded the available genomes from GenBank. The genomes were de novo annotated with the use of Prokka 1.14.6 [30]. For each genus, the resulting annotations were used to prepare five datasets for the Synima v 1.0 synteny imaging tool [31]. Four datasets were obtained by dividing all predicted protein-coding sequences from a particular genus into four equal parts according to their GC content, and the fifth dataset contained all CDSs. Synima was run with all five datasets independently and the resulting images were superimposed in a vector graphics editor. Data formatting was performed with the use of custom Python scripts, available at https://github.com/Ivan-Pchelin/scripts-for-synteny-visualization (accessed on 14 February 2022). Potential anti-CRISPR loci were predicted using AcrFinder [32]. The search for known antibiotic-resistance determinants and bacterial virulence factors was performed with the use of ABRicate v 0.8 [33] and its in-built databases ARG-ANNOT, CARD, NCBI AMRFinderPlus, Resfinder and VFDB [34,35,36,37,38].

### 2.7. Peritoneal Infection Model in Mice

Outbred male mice (n = 54) weighing 20–30 g were acquired from the laboratory breeding nursery of the Russian Academy of Sciences (Rappolovo, Leningrad Region, Russia). Two infection model experiments, one for each virus, were performed. In each case, the mice were divided into three equal groups. In the GVEsP-1 experiment, the groups numbered 8 animals. In the SSsP-1 experiment, each group numbered 10 animals. The *Enterococcus faecalis* strains CCUG 52538 (viral strain GVEsP-1) and Serg (viral strain SSsP-1) were used for modelling of the infection.

The animals were inoculated intraperitoneally with a lethal dosage of *E. faecalis*. The bacterial load was 2–4 × 10^9^ CFU for the SSsP-1 experiment and 3–5 × 10^9^ CFU for the GVEsP-1 experiment. In each experiment, the first group of mice did not receive treatment. The second group received 1 mL^−1^ 3 × 10^9^ phage stock per os 3 h after infection. The third group received an intra-abdominal injection of the same phage preparation 3 h after infection. The animal infection model experiment continued for 7 days. During the course of the experiment, lethal cases of infection were autopsied to check the circulation of bacterial pathogens and bacteriophages in blood and bloodstream organs (liver, heart, spleen). The organs were homogenized and cultured to detect viable enterococci. The obtained bacteria were tested for phage sensitivity. After seven days of the experiment, all surviving mice were executed and autopsied as described above. Statistical analysis of the experiment included estimation of the survival distributions by the Kaplan–Meier method and further comparison of the distributions by performing a logrank test in R 4.1.2 [39] with the use of the survival 3.3–1 package [40]. The survival curves were visualized in R using the survminer 0.4.9 package [41].

## 3. Results and Discussion

### 3.1. Isolation and Identification of Bacteriophages

The bacteriophage SSsP-1 was isolated from a sample of feces enriched with the *E. faecalis* strain Serg. The virus GVEsP-1 was isolated from a water sample from To Lich River (Hanoi, Vietnam) using the bacterial strain *E. faecalis* 5arctic. Taxonomic identification through MegaBLAST searches against viral sequences of the NCBI Nucleotide database placed GVEsP-1 in the *Shiekvirus* genus of the family *Herelleviridae*. The phage SSsP-1 clustered within the *Saphexavirus* genus of *Siphoviridae*. The identity of the studied genomes and their closest matches in the database was 92% for GVEsP-1 (compared to *Enterococcus* phage vB_EfaM_A2) and 77% for SSsP-1 (compared to *Enterococcus* phage vB_EfaS_IME198). Therefore, both bacteriophages belonged to undescribed viral species, given the currently applied 95% threshold [42].

From the early stages of research, the phages showed different behavior and different tropism to enterococcal strains. GVEsP-1 formed small colonies on a double agar layer. It was active against 61% of *E. faecalis* strains and 22% of *E. faecium* strains from our collection. On the double agar plates with sensitive cultures, the phage SSsP-1 initially formed smooth plaques. After a number of passages its plaques changed morphology, being surrounded by a pronounced halo zone. The phage SSsP-1 was active against 36% of our *E. faecalis* isolates. Neither phage infected other tested bacterial species, including *E. gallinarum*, *E. casseliflavus*, *E. hirae*, *E. durans*, *Staphylococcus* spp., *Streptococcus* spp., *Bifidobacterium longum* and *Escherichia coli*. In all cases of successful lysis of bacterial cultures, spot tests of serial dilutions revealed the presence of viable viral progeny. Known *E. faecalis* viruses were active against 7.6–70.5% of bacterial strains [43,44,45,46,47,48,49]. Therefore, the host range of GVEsP-1 can be considered broad, and the host range of SSsP-1 can be thought of as moderate.

Searches in the NCBI and the International Committee on Taxonomy of Viruses (ICTV) databases revealed the presence of one *Streptococcus* phage SP-QS1 with a *Streptococcus pneumoniae* host in the *Saphexavirus* genus [Almaghrabi et al., unpublished, GenBank accession NC_021868]. Formerly, enterococci were classified as group D streptococci by Lancefield [50]. In 1984, they were separated into their own genus after studies based on nucleic acid hybridization showed a more distant relationship to enterococci [51]. Therefore, we tested the activity of SSsP-1 and GVEsP-1 against streptococcal and staphylococcal strains. As mentioned earlier, the two phages did not infect any streptococcal or staphylococcal strains. *Bifidobacterium longum* and *Escherichia coli* are phylogenetically distant from *Enterococcus* and belong to Proteobacteria and Actinobacteria phyla, respectively. Given that ICTV considers host range as one of the criteria of taxonomic classification of viruses [52], the phages expectedly did not infect these strains.

### 3.2. Phage Life Cycle and Morphology

**Figure 1:**
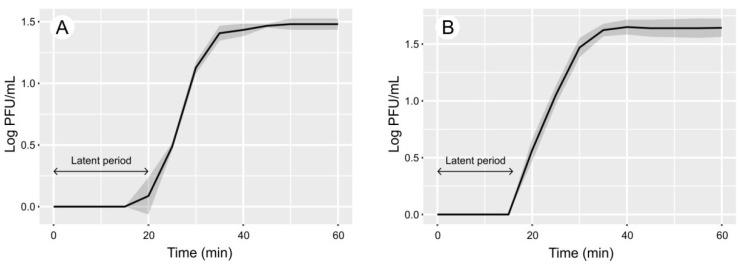
The length of the latent period in the development of bacteriophages assessed by one-step growth experiments. (**A**) *Enterococcus* phage GVEsP-1. (**B**) *Enterococcus* phage SSsP-1. The shaded areas delineate standard deviation in three biological replicates.

**Figure 2:**
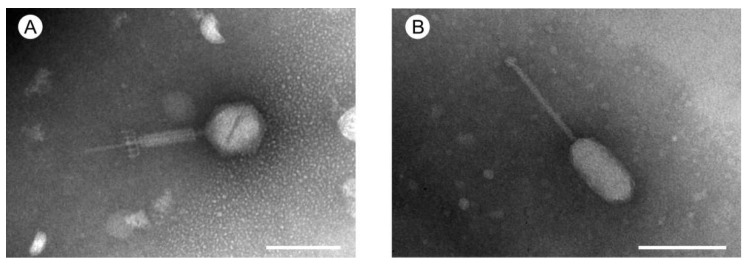
Morphology of the bacteriophages as revealed by transmission electron microscopy. (**A**) *Enterococcus* phage GVEsP-1. (**B**) *Enterococcus* phage SSsP-1. Scale bars = 100 nm.

The optimal MOIs for both phages were estimated at 0.01. One-step growth experiments for both phages did not show any prominent differences between *Saphexavirus* and *Shiekvirus* bacteriophages. In GVEsP-1, the length of the latent period was 20 min (Figure 1A). In SSsP-1, this phase took 18 min (Figure 1B). The average burst size for the phages SSsP-1 and GVEsP-1 was 66 ± 6 and 94 ± 4 PFU/cell, respectively. The GVEsP-1 phage possessed an icosahedral capsid with dimensions along the main axis of the particles of 77.8 ± 10.4 nm and dimensions along the cross axis of 77.7 ± 4.6 nm. A long contractile non-flexible needle-like tail in a sheath and a baseplate receptor under the sheath measured 160.5 ± 15.4 nm in length. No whiskers or legs were observed (Figure 2A). The capsid of SSsP-1 had an oval shape and was 87.7 ± 4.1 nm long, its width 43.2 ± 1.9 nm. The virus had a long, flexible tail measuring 117.5 ± 5.4 nm. A putative phage receptor was located on the baseplate (Figure 2B). These data were in agreement with a series of studies on other *Saphexavirus* and *Schiekvirus* viruses [44,53,54,55,56].

### 3.3. Genome Structure and Conservation

**Figure 3:**
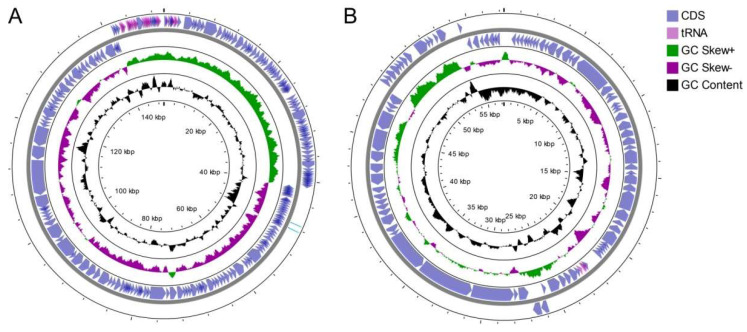
Genome organization of the bacteriophages. (**A**) *Enterococcus* phage GVEsP-1. (**B**) *Enterococcus* phage SSsP-1.

The GVEsP-1 genome sequence had a length of 149,913 bp with 194 predicted coding sequences, while the SSsP-1 genome measured 57,270 bp and contained 93 predicted CDSs. The GC content was 37% in GVEsP-1 and 40% in SSsP-1. The coding sequences in both genomes resided on the strands with positive GC skew (Figure 3). Additionally, both phages contained a cluster of genes coding for tRNAs. In the GVEsP-1 genome, there were 25 tRNA-coding genes, which is more than can be found in the genome of the closely related phage EFDG1. The SSsP-1 genome contained three tRNA genes. Neither phage carried integration-related genes and therefore they were obligately lytic.

**Figure 4:**
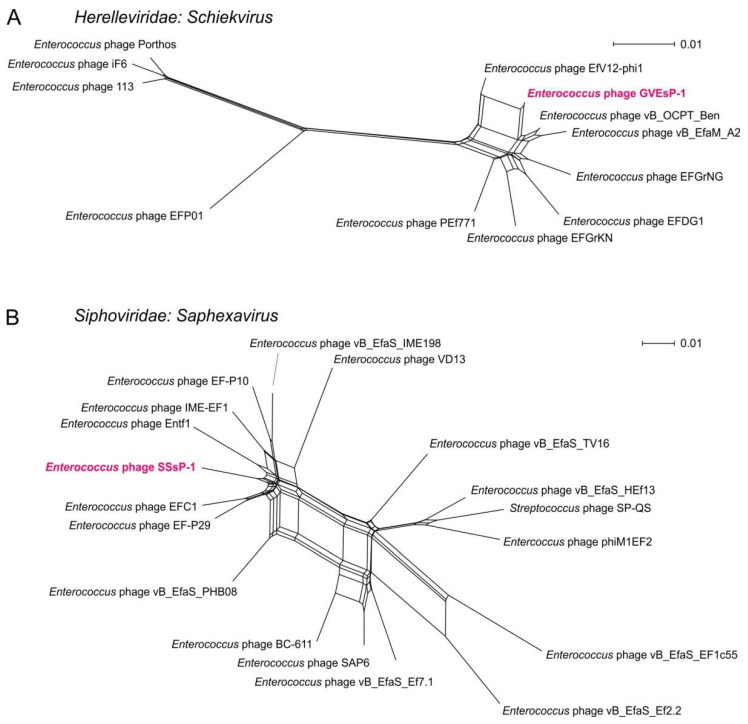
Evolutionary relationships of studied bacteriophages within *Schiekvirus* and *Saphexavirus* visualized by NeighborNet phylogenetic networks. (**A**) The position of GVEsP-1 within *Schiekvirus*. (**B**) The position of SSsP-1 within *Saphexavirus*. The viruses described in the present work are highlighted in purple.

To visualize the evolutionary relationships between the studied viruses within the genera, we calculated phylogenetic networks, using five protein-coding genes in both cases. The obtained alignments covered all the sequences available in GenBank for the *Shiekvirus* and *Saphexavirus* genomes. The *Shiekvirus* phylogenetic network had elements with tree-like structures, whereas in the *Saphexavirus* network, reticulation events prevailed (Figure 4). In both cases, the structure of the networks suggested either a significant impact of horizontal gene exchange in bacteriophage evolution [57] or the recent origin of the viruses from a common ancestor. The latter scenario may be supported by the high similarity of the phage SSsP-1 and the virus Entf1, isolated in the course of another Russian study (Rubalskii et al., unpublished, GenBank accession MK800154). However, recently diverged bacteriophage genomes are, at the same time, subjected to horizontal exchange of genetic information [58]. Still, a search for antibiotic-resistance determinants and virulence factors with the ABRicate tool in *Schiekvirus* and *Saphexavirus*, including the GVEsP-1 and SSsP-1 genomes, retrieved zero matches, which implies the safety of their potential clinical use.

**Figure 5:**
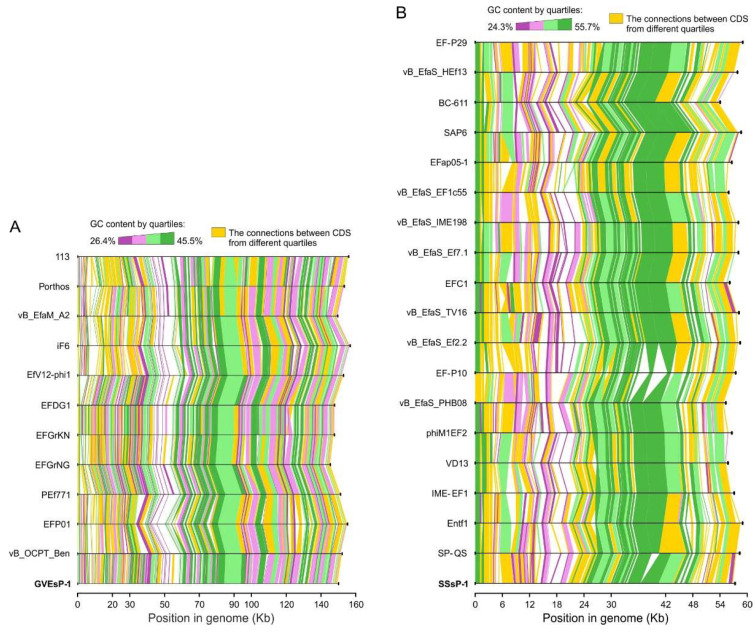
Protein-coding gene synteny in *Schiekvirus* (**A**) and *Saphexavirus* (**B**). The black axes correspond to the whole-genome sequences, whereas the colored connections indicate homologous coding sequences, determined by BLAST searches. All CDSs in the dataset were divided into quartiles according to their GC content. Color scale from purple to green indicates homology between the sequences within the same quartiles. The connections between CDSs from different quartiles are colored orange.

The whole-genome synteny in *Schiekvirus* and *Saphexavirus* revealed conservative sets and orders of protein-coding sequences, implying high degrees of structural conservation. The number of rearrangement events was neglectable (Figure 5). Therefore, the studied bacteriophages likely share a comparable biology, replication cycle and ecology with their congeneric viruses.

### 3.4. Potential Anti-CRISPR Loci

CRISPR–Cas systems provide a defense against heterologous genetic material, such as viruses, plasmids and other mobile genetic elements, for bacteria and archaea. They were first described in 2013 in several bacteriophages of *Pseudomonas aeruginosa* [59]. On the viral side, anti-CRISPR systems protect phage DNA through six theoretical mechanisms, including prevention of the insertion of viral DNA into the genome of a host cell, disruption of the synthesis of Cas proteins, blockage of crRNA synthesis, inhibition of crRNA loading onto Cas proteins, inhibition of DNA binding by Cas proteins and inhibition of DNA cleavage. The two latter mechanisms have been studied in vitro by molecular biology techniques [60]. The best-described anti-CRISPR proteins are known from the *Siphoviridae* phage family, myoviridae phages and prophages [59,61]. SSsP-1 and GVEsP-1 belong to siphoviridae and myoviridae morphotypes of obligately lytic bacteriophages, respectively.

In the SSsP-1 bacteriophage genome, most proteins identified by AcrFinder as potential anti-CRISPR loci did not have functional annotations. Still, in putative anti-CRISPR loci, pAcrS1, pAcrS3 and pAcrS4, there were proteins annotated as DNA-binding proteins and HNH homing endonucleases (Table 2). DNA-binding protein can probably block DNA binding by Cas. HNH homing endonuclease can be a protein with a HNH domain that is bound to the Cas protein HNH domain as it was described by Harrington et al. [62]. The anti-CRISPR loci in the GVEsP-1 genome were predicted with less confidence, so it was unclear whether they indeed had anti-CRISPR functions.

To sum up, well-known anti-CRISPR mechanisms interrupt CRISPR–Cas immunity at stages of expression and interference. *Enterococcus faecalis* is a regular carrier of a type II CRISPR–Cas system [63]. Well-known anti-CRISPR type II proteins exploit mechanisms of DNA-binding inhibition through binding to the HNH domain of Cas nucleases [62]. In the studied bacteriophage genomes, we found putative components of an anti-CRISPR system involving inhibition of DNA binding by Cas proteins. Potentially, the presence of anti-CRISPR proteins can make therapeutic enterococcal bacteriophages more efficient from a practical point of view.

### 3.5. Mouse Infection Experiments

**Figure 6:**
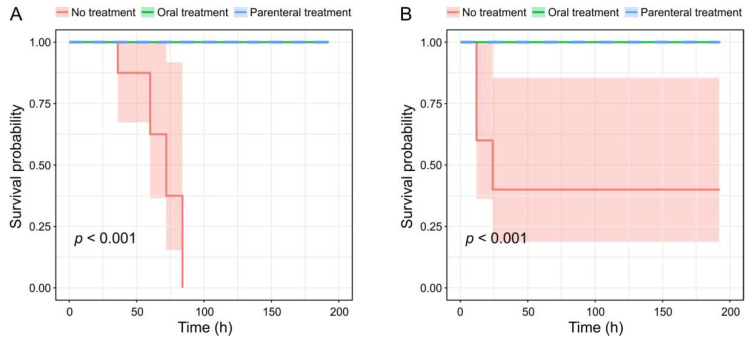
Survival curves in the animal infection model experiment. (**A**) Mice infected with *E.* *faecalis* strain CCUG 52538 and treated with the phage GVEsP-1. (**B**) Mice infected with *E.* *faecalis* strain Serg and treated with the phage SSsP-1.

In our peritoneal infection model experiments, untreated animals infected with *E. faecalis* CCUG 52538 died by the fourth day after inoculation. Six out of ten mice infected with *E.* *faecalis* Serg died by the end of the first day. All treated mice survived until the end of the experiments (Figure 6). Therefore, phage administration route did not affect the mortality rate of mice.

The differences in survival distributions between the treated and untreated animal groups were statistically significant (*p* < 0.001 by the logrank test), implying the potential of the studied bacteriophages for therapeutic applications [64]. The positive results of our in vivo experiment corroborate an earlier conclusion that enterococcal viruses from the families *Herelleviridae* and *Siphoviridae* are good candidates for phage therapy [65]. Our culture tests with blood and bloodstream organs of autopsied animals did not reveal the presence of viable phage particles. This can be explained by the deactivation of virions by the animal immunity system [66].

## 4. Conclusions

The high activities of two novel tailed bacteriophages against *Enterococcus* spp. were demonstrated. Both phages were able to protect mice from lethal enterococcal infection. The phages had conservative genomic structures and contained putative components of anti-CRISPR systems. Given the relative genetic stability of viruses from the genera *Saphexavirus* and *Schiekvirus*, the results of our animal infection experiment may indicate the suitability of studied bacteriophages for treating septic enterococcal infections.

## Figures and Tables

**Table 1:** *Enterococcus faecalis* strains used for experiments.

Isolate	Source
Serg	Urine of patient with urinary tract infection, Saint Petersburg, Russia
5arctic	Ornithogenic soil associated with *Rissa tridactyla*, Svalbard
ATCC 29212	Swedish Institute for Infectious Disease Control (SMI)
CCUG 52538	Swedish Institute for Infectious Disease Control (SMI)

**Table 2:** The putative anti-CRISPR loci detected by ArcFinder. The described version of the SSsP-1 genome is MZ333457.1; the version of the GVEsP-1 genome is MZ333462.1.

Phage	Putative Anti-CRISPR Locus	Strand	Number of ORFs	Start	End	Known ORF Annotations
SSsP-1	pAcrS1	-	10	529	3163	DNA-binding protein
SSsP-1	pAcrS2	-	9	5611	11,590	DNA-binding protein
SSsP-1	pAcrS3	-	2	14,071	14,808	HNH endonuclease
SSsP-1	pAcrS4	-	4	22,880	24,454	HNH homing endonuclease
SSsP-1	pAcrS5	+	2	25,842	26,812	HNH homing endonuclease
SSsP-1	pAcrS6	-	7	54,585	56,973	None
GVEsP-1	pAcrG1	+	2	4947	5568	DNA-binding protein
GVEsP-1	pAcrG2	+	11	22,501	26,731	Phosphoesterase
GVEsP-1	pAcrG3	+	6	30,688	32,712	None
GVEsP-1	pAcrG4	+	11	37,918	40,949	None
GVEsP-1	pAcrG5	-	3	52,269	53,288	None

## Data Availability

The nucleotide sequences for the phages GVEsP-1 and SSsP-1 have been deposited in the NCBI Nucleotide database under the GenBank/ENA/DDBJ accession numbers MZ333462 and MZ333457, respectively.

## Supplementary Materials

The following supporting information can be downloaded at: https://www.mdpi.com/article/10.3390/v14040831/s1, Table S1: Host range of GVEsP-1 and SSsP-1.
